# Venezuelan Equine Encephalitis Virus Infection of Spiny Rats

**DOI:** 10.3201/eid1105.041251

**Published:** 2005-05

**Authors:** Anne-Sophie Carrara, Marta Gonzales, Cristina Ferro, Margarita Tamayo, Judith Aronson, Slobodan Paessler, Michael Anishchenko, Jorge Boshell, Scott C. Weaver

**Affiliations:** * University of Texas Medical Branch, Galveston, Texas;; † Instituto Nacional de Salud, Bogotá, Colombia

**Keywords:** encephalitis, alphavirus, pathogenesis

## Abstract

Enzootic strains of Venezuelan equine encephalitis virus (VEEV) circulate in forested habitats of Mexico, Central, and South America, and spiny rats (*Proechimys* spp.) are believed to be the principal reservoir hosts in several foci. To better understand the host-pathogen interactions and resistance to disease characteristic of many reservoir hosts, we performed experimental infections of F_1_ progeny from *Proechimys chrysaeolus* collected at a Colombian enzootic VEEV focus using sympatric and allopatric virus strains. All animals became viremic with a mean peak titer of 3.3 log_10_ PFU/mL, and all seroconverted with antibody titers from 1:20 to 1:640, which persisted up to 15 months. No signs of disease were observed, including after intracerebral injections. The lack of detectable disease and limited histopathologic lesions in these animals contrast dramatically with the severe disease and histopathologic findings observed in other laboratory rodents and humans, and support their role as reservoir hosts with a long-term coevolutionary relationship to VEEV.

Venezuelan equine encephalitis (VEE) is an emerging disease that affected humans and equines in many parts of the Americas throughout the 20th century (1–6). The etiologic agent is VEE virus (VEEV), a positive-sense RNA virus in the family *Togaviridae* and genus *Alphavirus*. The first strain of VEEV was isolated and characterized serologically in 1938 (7,8). Numerous VEEV strains and closely related alphaviruses have since been classified into 2 epidemiologic groups: enzootic and epizootic strains. Enzootic strains (subtypes I, varieties D-F, and subtypes II-VI) are regularly isolated in lowland tropical forests in Florida, Mexico, and Central and South America, where they circulate between *Culex* (*Melanoconion*) spp. mosquito vectors and small rodents; these strains are generally avirulent for and incapable of amplification in equines (4,9,10). In contrast, epizootic VEEV strains (subtype I, varieties A-B and C), which are responsible for all major outbreaks in humans and equines, use several mosquito vectors and equines, which are exploited as highly efficient amplification hosts (11,12). Epizootic viruses cause debilitating disease with high fatality rates in equines. Humans are tangential, spillover hosts in both epidemic and enzootic VEEV cycles and are affected by most strains. A severe febrile illness that can occasionally be life-threatening develops; although human death occurs in <1% of infections with enzootic and epizootic VEEV strains, neurologic sequelae occur in survivors, particularly children (13).

Reservoir hosts play an important role in the replication, maintenance, and dissemination of arthropodborne viruses (arboviruses). These hosts generally show little or no disease after infection, presumably reflecting long-term selection for host resistance and possibly for virus attenuation (14,15). Changes in the habitats and ecology of reservoirs due to anthropogenic or natural causes can affect pathogen transmission to humans and domestic animals (16–18). Therefore, understanding how pathogens affect reservoir fitness, as well as how the reservoir affects pathogen replication and transmission, could facilitate prediction of emergence, reemergence, or extinction of sylvatic pathogens in response to environmental changes including deforestation. A better understanding of pathogen-reservoir interactions, particularly mechanisms of disease resistance, may also enhance the development of treatments for humans and domestic animals.

Field studies in Panama have identified antibodies to VEEV in many different species of mammals, including *Proechimys* spp. (spiny rats), *Sigmodon* spp. (cotton rats), *Marmosa* spp. (mouse opossums), *Didelphis marsupialis* (opossums), and Chiroptera (bats) (1,19–22). However, *Proechimys* spp. (family *Echimyidae*) and *Sigmodon* spp.(family *Muridae*) are thought to be principal reservoir hosts for enzootic strains because they are infected in nature, have high rates of immunity, and viremia develops in them after laboratory infection (20,23,24). These 2 rodents have different, but overlapping, geographic distributions; *Proechimys* spp. are found in Panama, northern Peru, Bolivia, Paraguay, and southern Brazil, whereas *Sigmodon* spp. are found from southern North America to northern parts of Venezuela and Peru. *Proechimys* spp. can be abundant in their forested habitats (25). They have a gestation period of 60 to 70 days and give birth to 2 to 3 pups per litter. Their natural life expectancy is ≈20 months and can exceed 2 years.

The relationships between rodent reservoir hosts and VEEV have received little study. Spiny rats (*Proechimys semispinosus*) captured in a VEEV-enzootic region of Panama exhibited ≈67% seropositivity (1). When spiny rats were infected with a local subtype ID VEEV strain, high-titer viremia developed, suggesting their role as reservoir hosts. Antibody was detectable by day 3 and persisted for up to 9 months (20). The role of spiny rats as VEEV reservoirs was reinforced by a study in Colombia and Venezuela in which a correlation was established between the abundance of these rodents and levels of enzootic circulation (10). Experimental infections of *Sigmodon* spp. also support their role as reservoir hosts, and horizontal transmission has been demonstrated among cage mates (20,23,24). However, none of these studies of spiny and cotton rats has investigated the clinical or histopathologic manifestations of VEEV infection in these reservoir rodents.

We examined interactions between VEEV isolates from an enzootic focus in the Middle Magdalena Valley of Colombia (10) and sympatric *P. chrysaeolus*. The lack of detectable disease and limited histopathologic effects on these animals contrast dramatically with the severe disease and histopathologic changes observed in other laboratory rodents and humans, and support their role as reservoir hosts with a long-term coevolutionary relationship to VEEV.

## Materials and Methods

### Animals

*P. chrysaeolus* (spiny rats) were obtained from a colony established at the Instituto Nacional de Salud, Bogotá, Colombia, from adults captured in the Monte San Miguel Forest in the Middle Magdalena Valley (10). The rats were identified using mitochondrial DNA sequence analysis and karotyping (J. Patton, University of California, Berkeley, CA, pers. comm.) (26). Male and female F_1_ offspring 3–36 months of age were used for experimental infections. The animals were housed in conventional rat cages and fed laboratory rat chow. All animals were tested for neutralizing antibodies against VEEV, and seronegative animals were infected in accordance with animal care and use guidelines of the Instituto Nacional de Salud. Organs were fixed for 48 h in 4% buffered formalin, embedded in paraffin, sectioned (5 μm), and stained with hematoxylin and eosin.

### Viruses

Enzootic subtype ID VEEV strain Co97-0054 was isolated in 1997 from a sentinel hamster in the same Colombian forest where the spiny rats originated (10). This virus was passaged once in baby hamster kidney 21 cells before animal inoculations. Enzootic strain 66637 was isolated in 1981 from a sentinel hamster in Zulia State, Venezuela (27), and had 1 passage in suckling mouse cells and 1 passage in African green monkey kidney (Vero) cells.

### Infections

Before infection, the animals were weighed and their body temperature was measured rectally. Animals were injected by the subcutaneous (SC) route into the left footpad with 3 log_10_PFU/mL of virus in a 50-μL volume, a dose consistent with alphavirus saliva titers in mosquitoes (28).

### Virologic and Histologic Tests

Infected animals were bled and weighed daily following ether anesthesia, and their body temperatures were recorded on days 1 to 4 and day 7. Blood samples were also collected from some animals at 1 month, 15 months, or both, postinfection. Blood was diluted 1:10 in Eagle minimal essential medium supplemented with 20% fetal bovine serum, gentamicin, and L-glutamine, and stored at –80°C. Viremia and levels of neutralizing antibodies were determined by plaque assay and 80% plaque reduction neutralization tests using Vero cells. For histologic analyses, 4 animals from days 1 to 4 postinfection and 2 from day 7 were killed and organs were collected. Samples containing virus and viral RNA from the heart, brain, liver, and kidneys of 2 animals killed on each of days 1–4 were homogenized and centrifuged for 10 min at 5,760 × *g*, and the supernatant fluids were stored at –80°C before virus titration or RNA extraction.

### Neurovirulence Studies

Four animals were injected by the intracranial (IC) route with 3 log_10_PFU of virus strain Co97-0054 following anesthesia with ketamine/xylazine (50/5 mg/kg). Animals were monitored for signs of illness, including loss of activity, ruffled coat, dehydration, anorexia, and neurologic disorder (erratic movements of legs or head), and bled daily for 4 days for viremia titration and on day 15 to determine seroconversion.

### Isolation and Amplification of RNA

RNA was extracted from triturated tissues with Trizol LS (GIBCO, Grand Island, NY, USA) following the manufacturer's protocol. DNA primers (5´-CGACAGAAAACCAGCAGAGACCTTG-3´, reverse primer: 5´-TCTAACATAGCCATCGTGCCCGTC-3´) were designed to amplify the VEEV genome at positions 8431–8677. cDNA was obtained by combining 1 μg of RNA in 10 mmol/L of dithiothreitol, 20 nmol/L of deoxynucleotide triphosphates (dNTPs), 4 μL of 5× first-strand buffer, 40 U of RNAse inhibitor, 100 ng/μL of minus sense primer, and water to give a final volume of 20 μL, and incubated for 2 min at 40°C. Two hundred units of reverse transcriptase (Superscript, GIBCO) were added, and the sample was incubated overnight at 42°C. A polymerase chain reaction (PCR) included 1 μg of cDNA, 300 ng of each primer, 10 μL of 10× Taq buffer (GIBCO), 1.25 mmol/L of Mg^2+^, 20 nmol/L of dNTPs, 5 U of Taq enzyme (GIBCO), and water to a final volume of 100 μL. Thirty cycles were performed, including denaturation at 95°C for 1 min, annealing at 59°C for 30 s, and extension at 72°C for 2 min, followed by a 10-min extension time at 72°C. PCR products were analyzed by electrophoresis on a 1% agarose gel, and DNA products were purified by using a QIAquick PCR purification kit (Qiagen, Valencia, CA, USA). PCR products were sequenced by using the sense primer and the ABI PRISM Big Dye Terminator v3.0 kit (Applied Biosystems, Foster City, CA, USA) following the manufacturer's recommendations.

### Statistical Analyses

Linear regression was used to analyze the influence of age on peak level of viremia. One-way analysis of variance was used to analyze tissue and viremia data. Body temperature differences were analyzed using the SAS/STAT procedure MIXED for repeated measures (SAS/STAT Users Guide, Volumes 1, 2, and 3, Version 8, SAS Institute, Cary, NC, USA).

## Results

### Clinical Manifestations

Twenty rats were infected with the enzootic ID strain Co97-0059, and 2 rats were infected with the enzootic strain 66637. All animals were monitored twice a day for clinical signs of VEEV infection, and none showed any signs of disease or discomfort, abnormal activity, disturbed social behavior, or loss of appetite, as compared with the uninfected control. No significant changes in body temperature were observed (p>0.25; data not shown). The 3 animals injected IC with strain Co97-0054 survived and showed no signs of illness. Of all animals infected either SC or IC (n = 27), only 1 showed clinical signs (hyperthermia) and died on day 3 postinfection. Whether death was due to VEEV or another cause is not clear. Histopathologic results from this animal are presented below. Viral titers in this animal were similar to those of others infected with the same virus.

### Viral Replication and Tissue Tropism

**Figure 1:**
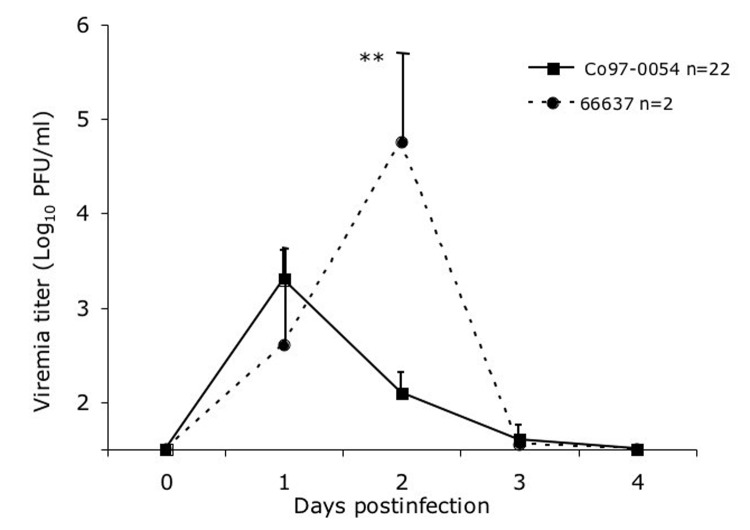
Viremia in spiny rats after subcutaneous infection with 3 log_10_ PFU of the enzootic Venezuelan equine encephalitis virus strains Co97-0054 and 66637. Vertical bars represent standard errors of the means. **p = 0.001.

In animals infected SC with the sympatric enzootic strain Co97-0054 peak viremia levels of up to 3.3 log_10_PFU/mL developed 24 h postinfection, but the virus was undetectable by day 4 (Figure 1). Similar viremia levels, and no detectable disease occurred in 3 spiny rats infected by the bite of an infectious mosquito (data not shown). No correlation between peak viremia titer and age could be established (n = 28, p = 0.08, R^2^ = 0.11, slope = –0.1). Animals infected with the Venezuelan enzootic ID strain 66637 exhibited a viremia level 1 log_10_PFU/mL higher viremia, with a delayed mean peak of 4.8 log_10_PFU/mL on day 2 postinfection; this difference in titer between virus strains was significant (p = 0.001). Clearance of the virus occurred at the same time, by 4 days postinfection (Figure 1).

**Figure 2:**
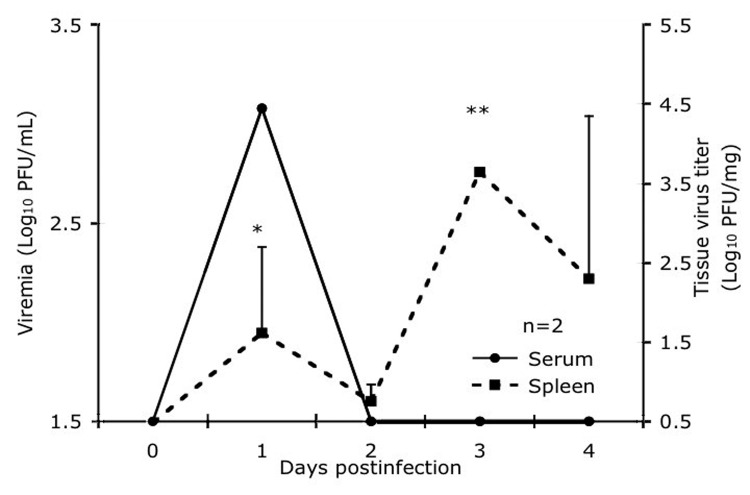
Comparison of the viremia titer with virus titer in the spleen in 2 spiny rats/time point after subcutaneous infection with 3 log_10_ PFU of enzootic Venezuelan equine encephalitis virus strain Co97-0054. Vertical bars represent standard errors of the means. *p = 0.04, **p = 0.0003.

Virus titrations were performed on the spleen, heart, brain, liver, and kidneys from 2 animals on each of days 1 to 4 postinfection. Only the spleen had detectable levels of virus on days 1 (mean 2.6 log_10_PFU/mg) to 4 (mean 3.9 log_10_PFU/mg), with a peak on day 3 (4.1 log_10_PFU/mg) (Figure 2). Peak virus titers in the spleen were reached 2 days later than with peak viremia level, and viremia was undetectable by day 4 when virus could still be isolated from the spleen. This finding suggests that the spleen is a major site of viral replication or clearance from the circulation after replication elsewhere. None of the other organs had detectable infectious virus during the peak viremia phase.

Because no VEEV was detected in organs other than the spleen, RNA extractions and reverse transcription–PCR, a more sensitive assay, were performed. The detection limit of the PCR was estimated at 0.3–1.3 PFU when virus stocks of known titer were used (29), while the detection limit of the plaque assay with the volumes available was 1.6 log_10_ PFU/mL. Viral RNA was detected in all organs tested, and sequencing of the PCR amplicons confirmed the presence of VEEV strain Co97-0054. However, detection of viral RNA does not necessarily indicate the presence of replicating virus in the organs because the animals were not perfused, and residual virus could have been present in blood.

Animals injected IC with the subtype ID strain had a peak viremia level 48 h postinfection, with titers from 2.6 to 3.2log_10_PFU/mL. Unlike SC infection, IC infection resulted in viremia levels for only 24 to 48 h (Table). The lack of detectable disease in these animals indicates that VEEV is not neurovirulent for these reservoir hosts.

**Table:** Viremia and neutralizing antibody titers of spiny rats infected intracerebrally with the Co97-0054 strain of Venezuelan equine encephalitis virus

Rat no.	Viremia*				Neutralizing antibodies
	Day 1†	Day 2†	Day 3†	Day 4†	Day 15†
105	<0.85	2.94	<0.85	<0.85	1:320
107	<0.85	<0.85	2.20	<0.85	1:320
126	3.20	3.2	<0.85	<0.85	1:640
74	<0.85	2.6	<0.85	<0.85	1:1,280

*Viremia levels are expressed in log_10_ PFU/mL; 0.85 log_10_ PFU/mL was the detection limit. †Day postinfection.

### Antibody Development

Neutralizing antibodies developed in all animals by day 7 postinfection, with a mean titer of 1:160. One year postinfection, antibody titers for surviving animals were 1:20–1:640. Detectable viremia did not develop in animals rechallenged with VEEV 1 year postinfection, but the animals did exhibit an 8-fold increase in antibody titers. All animals infected IC had detectable neutralizing antibodies 15 days postinfection (1:320–1:1,280).

### Histopathologic Analysis

**Figure 3:**
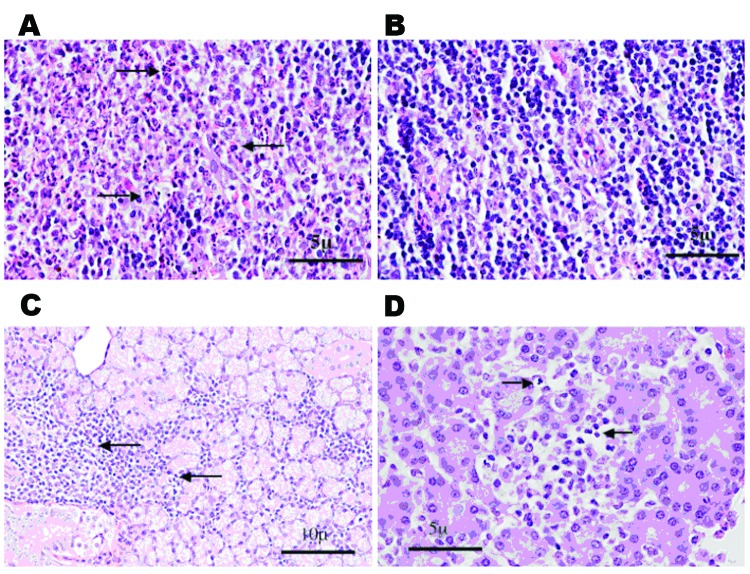
Histologic staining (hematoxylin and eosin) of spiny rat lymph nodes (A and B), salivary glands (C), and pancreas (D) after subcutaneous inoculation of 3 log_10_ PFU of Venezuelan equine encephalitis virus strain Co97-0054. A) Popliteal draining lymph node 24 h postinfection, showing the presence of a polymorphonuclear leukocyte infiltrate (arrows). B) Contralateral popliteal lymph node 24 hr postinfection from same animal. No proinfiltration was visible. C) Chronic inflammation of the salivary gland (arrows) day 3 postinfection. D) Asenea with focal necrosis (arrows) day 3 postinfection. (Magnification ×40.)

Histopathologic analysis was performed on small numbers of SC-infected animals on days 1 to 4 and day 7 postinoculation. Overall, pathologic changes were mild. All 4 animals examined at day 1 showed an acute lymphadenitis in the draining, left popliteal lymph node. This lesion was characterized by infiltration of the subcapsular sinus and cortical follicles with neutrophils, with minimal necrosis (Figure 3A and B). This lesion was not seen in the contralateral popliteal lymph node or in inguinal lymph nodes and disappeared by day 2. No splenic lesions attributable to VEEV were seen at any time. A striking degree of hemosiderosis of the red pulp, along with mineralization of the capsule and trabeculae, occurred in many animals, including uninfected controls, and appeared to be a normal, age-related phenomenon in this species. No brain or meningeal inflammation was seen at any time. Lesions of other organs were sporadic, including interstitial or periductal chronic inflammation in the salivary glands (Figure 3C) and multifocal chronic inflammation in the pancreas. Inflammatory foci were rarely seen in the heart on days 1, 4, and 7 but were not seen in controls. The kidney and thymus showed no lesions. Pathologic changes of the viscera appeared to peak on day 3, when 2 of 3 rats killed showed salivary gland pathology, 2 showed lung pathology, 1 showed pancreatic pathology (Figure 3D), and 2 showed liver pathology.

One spiny rat had hypothermia and weight loss 24 h after infection and died on day 3 postinfection. Necropsy showed extensive liver pathology, characterized by steatosis, and confluent coagulative necrosis with a mixed, mononuclear cell–predominant inflammatory infiltrate. This animal also showed pulmonary edema and striking alveolar hemosiderosis. Another animal exhibited alveolar edema and patchy acute inflammation in the lung 3 days after infection, while a different animal in this group displayed periportal acute inflammation in the liver. These lesions were not seen in other animals. The lung histopathologic pattern seen in 2 animals on day 3 postinfection suggests that it might be VEEV-related; however, we cannot rule out other processes in these colony-reared animals.

## Discussion

Subcutaneously VEEV infection induced little or no disease in *P. chrysaeolus*, the principal reservoir host in central Colombia. No signs of disease were seen after intracranial injection and at least 96% of animals showed no clinical signs after SC infection. Viremia was of low magnitude and short duration after SC inoculation, and was almost completely lacking after IC infection. Seroconversion occurred by day 7 and was persistent for at least 1 year. Histopathologic changes suggestive of viral cytopathic effects were visible in the draining lymph nodes 24 h postinfection and by day 3, mild pancreatic pathology was visible. The inability to detect infectious virus in the brain indicated that VEEV in these rats is probably not neuroinvasive; even after IC inoculation, detectable disease did not develop in any rat, indicating that VEEV is not neurovirulent in these animals. The only organ tested that showed evidence of viral production was the spleen; although viral RNA was detected in all other organs, blood contamination could not be ruled out.

In SC-infected spiny rats detectable levels of viremia developed for 3 days. The difference in viremia level between the 2 enzootic strains has been reported for another VEEV reservoir host, the cotton rat, using 2 strains of Everglades virus (30). Strain variation could explain the higher viremia titers reported for Panamanian spiny rats infected with Panamanian VEEV (20) as compared to our results with Colombian strains and animals. Other factors that could explain this difference include 1) the Panamanian virus used previously had a higher passage history than our strain Co97-0054 (3 suckling mice versus 1 baby hamster kidney cell) that could have led to adaptation for rodent replication (31); and 2) different species of *Proechimys* from a different locality were used in the previous studies.

Although the viremia titers we measured in spiny rats were lower than those generated in laboratory mice (3–4 log_10_ versus 6–7 log_10_PFU/mL), they are sufficient to infect enzootic mosquito vectors that have been shown to be highly susceptible to infection by enzootic strains of VEEV (12,32,33). Furthermore, *Culex* (*Melanoconion*) *pedroi*, *Cx*. (*Mel*.) *spissipes*, *Cx*. (*Mel*.) *vomerifer*, *Cx*. (*Mel*.) *crybda*, and *Psorophora albipes* mosquitoes, some of which are natural VEEV vectors (12), captured in the Monte San Miguel forest became infected after feeding on viremic spiny rats (Carrara AS and Ferro C, unpub. data).

No virus was found in the feces (data not shown). Although saliva was not sampled, a previous study reported virus in throat swabs of both spiny rats and cotton rats (20). The pathologic changes we observed in the salivary glands, although nonspecific, suggest the possibility of VEEV infection in this site. Other studies have shown that VEEV is horizontally transmitted among rodents (23); in spiny rats, this transmission might occur orally during social contact and probably not through urinary and feces contamination. Further experiment designed specifically to address this issue are needed.

Virus titers in the spleen suggest it is a principal site of viral replication in spiny rats. The draining lymph node, as suggested by the presence of viral antigen and the high level of neutrophil infiltration 24 h postinfection but absent after 48 h, may be a site of initial viral replication as in mice (34). Viral RNA detected in other organs indicates either a very small amount of replication (below the plaque assay detection limit) or the presence of RNA in viremic blood. The involvement of the pancreas as a target for VEEV replication during the later stages of infection is reminiscent of similar findings for TC-83 (attenuated VEEV vaccine strain) infection of mice and hamsters (3). In mice, VEEV also disseminates to the spleen after initial replication in the draining lymph node (35). However, an important difference is that enzootic VEEV appears to be nonneuroinvasive and nonneurovirulent in spiny rats, in contrast to its uniformly neuroinvasive and neurovirulent phenotype in laboratory mice.

Neutralizing antibodies were detected in spiny rat serum from 7 days to 1 year after VEEV infection. These antibodies apparently prevent reinfection by homologous (subtype ID) or heterologous (subtype IC, data not shown) VEEV subtypes 1 year after infection. Therefore, spiny rats appear unlikely to be susceptible to reinfection. Based on human data after vaccination with the TC83 strain, neutralizing antibodies are even longer lasting (36).

Seroconversion of spiny rats in nature may present a limiting factor to VEEV circulation, where the virus depends on the constant generation of naive rodents. Their long gestation and small litter sizes suggest that other reservoir animals may be required to maintain enzootic VEEV transmission when spiny rat populations are low (10). Further studies are needed to investigate other possible reservoir hosts, such as bats and opossums, which have also been shown to be seropositive in nature (12).

The high survival rates found in these rats after either enzootic or epizootic VEEV infection (A.S. Carrara and C. Ferro, unpub. data) support previous conclusions of their role as reservoir hosts (37). By comparison, laboratory mice (*Mus musculus*) exhibit a 100% mortality rate with subtype ID strains of VEEV, including those we tested (6). Horses have low mortality rates with enzootic strains, but deaths from epizootic strains can exceed 50% (11). Human mortality rates are generally <1% with both enzootic and epizootic VEEV strains, but severe neurologic disease develops in 4% to 14% of VEEV-infected patients <15 years of age; the mortality rate in these patients can reach 20% (38).

Not only did spiny rats survive VEEV infection (96%), but they also showed little sign of illness or discomfort, and their social behavior and fertility were not appreciably altered by infection. No significant change in the fecundity of infected females was observed compared to uninfected colony animals (p = 0.86, Student *t* test). In contrast, laboratory mice infected even with the attenuated VEEV vaccine strain TC-83 exhibit aggressive behavior with cage mates (39). The high survival rate of spiny rats in spite of abundant replication suggests selection for resistance to disease. The spiny rat is part of a very old (≈25 million years) family (*Echimyidae*) (40), and long-term exposure to VEEV may have selected for resistance to disease in these animals.

Another possible explanation for the lack of disease in spiny rats is that VEEV has been selected for attenuation in these reservoir hosts. Other viruses, such as myxoma, have been shown to adapt to their hosts through attenuation. After its introduction into Australia for controlling the imported European rabbit population, myxoma virus underwent attenuation with concurrent selection for resistance in the rabbit population (14).

Spiny rats may be a useful tool for studying VEE pathogenesis and mechanisms of natural resistance. Additional studies of the immunologic responses in these rodents, particularly innate immunity, may provide valuable information that could be used to develop improved therapeutics for human and equine VEE.
